# Talent identification and recruitment in youth soccer: Recruiter’s perceptions of the key attributes for player recruitment

**DOI:** 10.1371/journal.pone.0175716

**Published:** 2017-04-18

**Authors:** Paul Larkin, Donna O’Connor

**Affiliations:** Sydney School of Education and Social Work, University of Sydney, Sydney, New South Wales, Australia; Universidade de Tras-os-Montes e Alto Douro, PORTUGAL

## Abstract

Using the modified Delphi method, we aimed to understand the attributes youth coaches and recruiters perceive as important when identifying skilled youth performance at the entry level of representative soccer in Australia (i.e., Under 13 years). Furthermore, we also aimed to describe the current methods youth coaches and recruiters use to assess and identify these attributes in youth players. Australian regional youth technical directors and coaches (n = 20) completed a three stage process, including an initial interview and two subsequent questionnaires, whereby attributes and qualities associated with talent identification were rated and justified according to the importance for youth player performance and talent identification. Results indicate a hierarchy of attributes recruiters perceive as important for Under 13 soccer performance, including technical (i.e., first touch, striking the ball, one-versus-one ability, and technical ability under pressure), tactical (i.e., decision-making ability) and psychological attributes (i.e., coachability and positive attitude). In addition, the findings indicated attributes and qualities not emphasised within the talent identification process including, physiological, anthropometrical, sociological and several psychological attributes. It is suggested talent recruiters apply a holistic multidisciplinary approach to talent identification, with the current findings potentially providing initial evidence to suggest recruiters do consider numerous attributes when selecting and identifying youth players.

## Introduction

Within sports, talent selection and identification are imperative for the development of future elite level performers; however the process is complex with many coaches using numerous physiological and technical performance assessments to identify future elite players [1, 2]. Identification of the next generation of sports stars is an important aspect of a youth coach’s role. Early talent identification research suggested the process of identifying talent was based on genetic or innate predispositions that may be responsive to training intervention [3]. Talent identification has traditionally been based on viewing athletes in a trial game or training session environment, whereby the players aim to impress coaches. This approach to talent selection or recruitment is not informed by scientific evidence, but rather coaches subjective preconceived notion of the ideal player, which used in isolation may result in repetitive misjudgements and limited consistency [4, 5]. Therefore, it is of interest to further investigate this underrepresented area in the talent identification literature to gain a better understanding of the possible attributes and strategies used by coaches and recruiters when identifying potentially talented players.

The central premise of talent identification and recruitment is to identify and select the most promising young athletes with the potential to excel and become a successful professional senior athlete [6, 7]. In team-based sports, such as soccer, talent identification is a complex process due to the different qualities associated with performance, including physical, physiological, technical and tactical attributes, as well as psychological and sociological influences [3, 6–8]. The challenge for coaches and recruiters during the talent identification process is that these qualities are dynamic, interact with one another, and are responsive to practice and training [6–7]. As successful soccer performance is a complex interplay of multiple interacting skills and qualities, it is recommended youth soccer coaches should consider a holistic multidisciplinary approach to talent identification, rather than isolated assessments of individual skills and qualities [3, 8]. Despite this recommendation there is limited exploration of the link between research knowledge and current applied talent identification and recruitment practice. Therefore to progress the current knowledge it is important to understand the perceptions and observations of youth coaches and recruiters, in particular the player attributes they currently consider important (i.e., their main focus) and how they identify them in players when attempting to predict or identify potential future elite players.

To inform the talent identification and recruitment process, there is an extensive body of research exploring the skills and qualities that may discriminate skilled and less-skilled performance at a youth level (i.e., 11–17 years), including anthropometric and physiological [1, 9], perceptual-cognitive [10–12], and psychological factors [10, 13]. With respect to anthropometric and physical performance measurements, researchers indicate skilled youth soccer players are heavier, taller and faster than matched less-skilled players [9, 14–15]. Furthermore, researchers have identified relative age effects at elite youth competitions [16–17], with players born in the first quarter of the year more likely to be perceived as more talented due to their physical advantage compared to players born later in the selection year due [18]. This demonstrates that coaches may be influenced by physiological measurements when recruiting youth players.

Technical skills such as passing, first touch and dribbling have been found to constitute over half of all the individual actions performed within a game [19], with research indicating teams who maintain possession of the ball are more likely to be successful [20–21]. Therefore, there is great importance placed on the technical ability of players, with a substantial body of research indicating technical skills differentiate youth soccer performance [1, 9, 14, 22]. Researchers have found players who progressed to an elite level of participation were more technically competent for skills such as ball control (i.e., ability to keep the ball in the air without using the arms or hands), dribbling speed and passing accuracy [14]. However, it should be noted that the majority of the investigations base their findings on the assessment of isolated dribbling performance (i.e., drill-based tests) which has been found to contribute to approximately 8% of all in-game individual actions, compared to first touch and passing which accounts for 46% of all in-game actions [19].

In addition to physiological and technical attributes, researchers have also indicated skilled youth players possess greater domain-specific information processing abilities such as decision-making, anticipation, situational probability and pattern recognition [11–12]. This extant knowledge base outlines that skilled players possess superior perceptual-cognitive skills when compared to lesser skilled players. While this provides an indication of the attributes that can differentiate skilled performance, there is still limited understanding of whether coaches value these attributes, and if so, how do they identify perceptual-cognitive skills in skilled youth players.

Further, researchers have indicated psychological attributes such as self-confidence, motivation, mental toughness, commitment, and seeking social support, may predict elite level soccer career success [5, 23–24]. In addition, models outlining the potential predictors of talent in soccer highlight the need for the consideration of sociological factors such as parental support, cultural background and hours of practice [5, 25]. Despite these findings there is still limited acknowledgement of these qualities within the talent development and recruitment process, however researchers indicate inclusion of psychological and sociological qualities within this process may provide a more holistic description of potential talent [5, 25–26].

In soccer, youth coaches and recruiters are continually seeking the attributes and qualities that may predispose individuals for a successful soccer career [14, 27]. While it is recommended that coaches and recruiters consider a holistic multidisciplinary approach to talent identification [3, 8], there is still limited understanding of how they conceptualise ability to identify future talent. Therefore, we aimed to understand what attributes youth coaches and recruiters perceive as important when identifying skilled youth performance at the entry level of representative soccer in Australia (i.e., Under 13 years). This age group is important to explore as in Australia Under 13 representative teams are the first step in the talent development pathway, with possible selection for these teams resulting in more opportunities for training and development and further national development programs. Furthermore, we also aimed to describe the current methods youth coaches and recruiters use to assess and identify these attributes in youth players.

## Materials and methods

### Study design

The Delphi method is a structured interactive communication process aimed at the collection of anonymous knowledge and judgements of experts to generate a consensus of opinion via the administration of repeated questionnaires with justified responses [28–30]. Thus, the Delphi method provides more in depth and reliable analysis of the research questions and is more effective than a single interview or questionnaire [28]. For the current study the modified Delphi method [28–29] was adopted. This design entailed an initial interview followed by two rounds of questionnaires.

### Participants

The Delphi method requires purposeful sampling of a small panel of domain-specific experts to achieve reliable responses, as a result a strict selection criterion was enforced for participant selection [28–29, 31]. Participant selection was based upon the unique perspectives and experiences in elite youth soccer coaching within Australia. All participants were either the youth technical director or head coach of an Australian regional representative team competing at the Under 13 national soccer championships, which is the highest level of competition for Under 13 players in Australia. The Under 13 level was chosen for this study as this is the first age level in Australia where players are selected for representative teams to compete at a national age-related championship in front of national recruiters. Eight regional technical directors and twelve head coaches agreed to participate in the study. On average the technical directors, who all hold a current Asian Football Confederation (AFC) / Football Federation Australia (FFA) Pro coaching license, had been involved in youth player identification and development positions for 14.6 (± 9.3) years, and employed in their current role for approximately 6.25 (± 4.8) years. The coaches had been coaching representative youth teams for 10.9 (± 5.4) years, and regional Under 13 representative team coach for approximately 5.3 (± 3.4) years. Within the coaches group, one coach had an AFC/FFA Pro License, two held an AFC/FFA Level A coaching license, one held an AFC/FFA Level B coaching license, and eight currently held an AFC/FFA Level C coaching license. Ethics approval was granted by the University of Sydney’s Human Research Ethics Committee. All participants were informed of the study procedures before providing written consent prior to participation.

### Procedures

#### Initial interview

To achieve a complete profile of the attributes associated with skilled Under 13 soccer players, an inductive qualitative semi-structured interview was chosen as the initial method of inquiry. Similar to previous studies [28–29], this approach allows participants more scope to develop a rationale for their opinions through conversation. Open-ended questions promoted consistent discussion to identify the key attributes participants value when identifying talented youth players for selection into a regional representative team to compete at the national championships, including technical, physiological, anthropometric, psychological and tactical attributes (e.g., when you are identifying talent at an Under 13 level what is it you are looking for?). Probing questions were also incorporated to understand why the participant thought the attribute was important (e.g., why is this attribute important when identifying Under 13 level players? Are there some attributes you believe to be more important than others when identifying talent Under 13 players?), an example of the attribute (e.g., can you provide an example of the attribute from a game context?) and how the participant identifies the attribute in players (e.g., can you provide an example of how you would identify this attribute in a player? How do you decide between talented or less talented players at this age group?) (See S1 File). As the current player development strategy in Australia indicates that up to and including the Under 13 age group players should be allowed and encouraged to play in multiple playing positions, participants discussed attributes in a general context, rather than position specific.

Interviews were conducted in a one on one manner face to face and ranged in length from 30 to 45 minutes, and were facilitated by the first author. Similar to Cupples and O’Connor [28], open coding was conducted to identify meaning units (i.e., sentences or ideas that described a specific attribute) from the data [32]. Following this, a panel of three experts (i.e., two coaching researchers and a youth coach with a Level C coaching license and Masters in Sports Coaching) discussed in a round table forum the grouping of similar meaning units. Any discrepancies in the grouping of meaning units were discussed until 100% agreement was reached, with this process identifying an extensive list of 29 player attributes (e.g., decision-making, first touch, strength). Finally, the expert panel operationally defined each attribute; for example, the operational definition for the attribute x-factor included, unpredictable, creativity, thinks outside the box (see Table 1).

**Table 1:** The operational definitions of each attribute as identified by meaning units provided by the participants during the interview process.

Attribute	Definition
**First Touch**	One of the four core skills outlined by FFA National curriculum; good touch when receiving the ball; good first touch; ability to control the ball; understanding of how the ball rolls
**1 v 1**	One of the four core skills outlined by FFA National curriculum; comfortable in attacking 1v1 situations; taking on an opponent
**Striking the Ball**	One of the four core skills outlined by FFA National curriculum; ability to pass the ball; accuracy of the pass; can play a disguise pass; pass their way out of trouble; strikes a ball in more ways other than the inside of his foot; confidence using both feet
**Coachability**	Ability to be coached; willing to learn; coachable; good learners; responsive to coaches
**Decision-making**	Make good decisions; smart; football brain; intelligent; identify options, accurately play the best option, thought behind the intention; can identify where to take the ball
**Positive Attitude**	Positive reaction after a mistake; how they handle disappointments; resilience; ability to overcome adversities; not wanting to give up
**Technique Under Pressure**	Good technique under pressure; can keep the ball under pressure; first touch in tight areas; doesn't panic on the ball
**Running with the ball**	One of the four core skills outlined by FFA National curriculum; can they run with the ball and maintain control
**Game Sense / Awareness**	Game awareness; football awareness; read the game; ability to scan; perception; tactically aware; aware of their surroundings
**Love of the Game**	Buy in to football culture; watches football; knows football; self-educating by watching; loves the game; training themselves; putting extra work in; fans of the game
**X-Factor**	Unpredictable; creativity; thinks outside the box; talent; gift
**Anticipation**	Identify early, intuition, foresee movements, can see a pass, knows what to do before they receive the ball, knows where to defend without advice and before it's occurred
**Confidence**	Confident within a group; brave; wants to be involved; wants the ball; wants the ball under pressure; confident to be able to get into positions to receive the ball all the time; have the guts to try and fail and do something different; belief in themselves; no fear of failure
**Competitive**	Resolve; desire; hunger; strong willed; determination; intense; fighting approach towards wanting the ball; winning mentality
**Consistent Execution**	Being able to perform skills and action well consistently; precision; execution; comfortable with the ball
**Personality / Character**	Disciplined; hard worker; wants to win in the right way
**General Game Understanding**	Understand the game tactically; try to create options; finds space; constantly moving; smart off the ball; correct positioning; support in the right place; being in the right place at the right time
**Vision**	Ability to scan the game area and see important information; Can use peripheral vision to see what is around them
**Adaptability**	Adapt skills to game situations; how they react to information given to them
**Agility / Balance**	Explosiveness, quickness, change of pace, change of direction, mobility, good movements, balanced running with the ball
**Team Understanding**	Can affect the game; effective in the game; understand positions and their roles; tries to do what he's been asked to do; adapt to more than one position; can play in different positions
**Concentration**	Ability to focus during games and training
**Defensive ability**	Defend 1v1 situation effectively; strong defensively
**Professionalism**	How they manage themselves; how they carry themselves on and off the pitch; contribute to the team environment; self-analyse; growth mindset; willingness to accept that everything is a challenge
**Communication**	Can have a dialogue with players and coaches; talks during the game; ability to listen to both players and coaches; have positive interactions with peers; prepared to ask questions of players and coaches; appropriate body language
**Speed**	Pace, speed, quick reaction, fast running with the ball
**Pressure**	Ability to deal with game-related pressures
**Strength**	Strikes the ball with power and over greater distance; core body strength
**Short Stature**	Shorter, smaller, little

#### First round questionnaire

The first round questionnaire included all 29 attributes identified during the initial interview. Participants were provided with instructions for the completion of the questionnaire, and the operational definitions of each attribute. During this stage, each attribute was evaluated, rated, rationalised, modified, or deleted by the participants. Participants rated the attributes according to the Miller’s Scale Battery of International Patterns and Norms [33], which provides an indication of degree of importance for each attribute listed on a scale from 0–9. The scale uses three main anchor points of reference with a bandwidth of three points between each anchor, least important (i.e., 1–3 points); moderately important (i.e., 4–6 points); and most important (i.e., 7–9 points). Any attribute participants believed should be deleted was given a score of 0. Participants were also encouraged to provide a justification for their rating for each attribute. All 20 participants correctly completed the first round questionnaire (i.e., provided a rating and justification).

Following the completion of the first round questionnaire, responses were recorded with mean group ratings calculated for each attribute, with 17 attributes obtaining a mean rating of 6 or above (i.e., top anchor of the moderately important band and the entire most important band). These 17 attributes formed the second round questionnaire.

#### Second round questionnaire

The second round questionnaire asked the participants to review, rate and justify the updated list of 17 attributes. Participants were informed the presentation of the attributes was based on the ranking from the previous round with the highest mean ranked attribute presented at the top of the questionnaire; however information relating to the justification for the ranking were not presented. By presenting the attributes in this high to low manner, participants are able to reflect on the overall group rankings compared to their responses [31], but are not influenced by others justifications. Participants were informed this may be the final round of the process (if consensus was reached) and thus the last opportunity to provide a justification for their ranking of the attributes. Overall, 18 of the 20 participants correctly completed the second round questionnaire (i.e., provided both rating and justification).

As the order of importance of the 17 attributes did not change between the first and second round questionnaire, a stable level of consensus was determined between participants. Data analysis determined the ranking or order of importance of the key attributes based on the mean and standard deviation. The justification for the ranking provided by the participants in the questionnaire was also compiled. Example of the justification that best reflects the participant’s perceptions are provided to support all numerical values [29].

## Results

As there were no differences between technical directors and coaches, all results were combined for analysis purposes. Table 2 provides the associated ranking scores of the attributes, and attributes deleted by the participants. Based on the analysis of the participant responses, a hierarchy of attributes exists with seven attributes identified as most important to performance and eleven attributes moderately important to Under 13 performance (see S1 Dataset). Analysis indicated emergent attributes from key performance categories, including technical, tactical and psychological. To facilitate interpretation, participants were assigned pseudonyms, with technical directors represented with the letter *T* (e.g., Thomas) and coaches with the letter *C* (e.g., Campbell).

**Table 2:** Mean ratings and standard deviations of the attributes associated with elite soccer performance at the Under 13 level following the second round questionnaire.

	Attribute	Mean Rating	SD
Most Important	First Touch	8.50	0.82
	1 v 1	7.88	1.02
	Striking The Ball	7.63	1.02
	Coachability	7.38	1.09
	Decision-making	7.38	1.09
	Positive Attitude	7.25	1.06
	Technique Under Pressure	7.00	1.65
Moderately Important	Running With The Ball	6.94	1.00
	Game Sense/Awareness	6.94	1.39
	Love Of The Game	6.88	2.70
	X-Factor	6.63	2.80
	Anticipation	6.60	1.50
	Confidence	6.56	1.71
	Competitive	6.38	1.89
	Consistent Execution	6.07	2.31
	Personality/Character	6.00	2.61
	General Game Understanding	5.79	2.36
Deleted after First Round	Vision		
	Adaptability		
	Agility/Balance		
	Team Understanding		
	Concentration		
	Defensive Ability		
	Professionalism		
	Communication		
	Speed		
	Pressure		
	Strength		
	Short Stature		

Evident from the data is the importance of technical skill proficiency, with first touch (i.e., the ability of the player to control the ball at initial ball contact), one-versus-one ability (i.e., the ability of the player to perform actions to get past an opponent), striking the ball (i.e., the ability of the player to distribute the ball), technique under pressure (i.e., the players ability to perform technical skills in pressured situations) and running with the ball (i.e., the players ability to control the ball while running) all rated as important attributes. Of these attributes participants ranked first touch as the most important, indicating it is “*the most important technical tool*” (Campbell) because for a player *“quality first touch is fundamental*, *as it is on most occasions the start of all soccer actions such as first touch to pass*, *first touch to run with the ball*. *Therefore it is a very important skill”* (Colin). This is reinforced by Clive who stated, “*it is a vital attribute*, *because without a good first touch players are unable to maintain possession and the other core skills of the game are not possible*”.

Participants indicated one-versus-one ability was an important performance indicator as it can “*assist attacking play to create and score goals*” (Caden). Further, this attribute was also believed to be crucial when selecting players, as “*the ability of a player to have the confidence and creativity to beat a direct opponent at speed in a one versus one situation sets them apart*. *Therefore*, *it is an extremely important factor when selecting a player*” (Colin). While these statements relate to the attacking dimension of a one-versus-one situation, only one participant indicated “*… but players who are effective in both sides of a one-versus-one (attacking and defending) are even better*” (Callum).

The third ranked attribute was striking the ball, with participants indicating “*players need to be able to strike the ball*, *ideally with both feet*” (Thomas), and it is an “*essential attribute as soccer is a passing game*” (Caden). This is further justified by Cameron who states “*For me*, *with my philosophy on soccer*, *if you can’t pass you can’t play*. *So I look for players who can play a good pass*. *When you break it down*, *it is not just passing the ball from one player to the other; there is speed*, *accuracy and selection of the pass”*. Participants also indicted the maintenance of a player’s technical performance during pressured game situations was a key attribute stating it is “*important to be able to produce an action in match conditions and with increased pressure*” (Corey) or “*Do they lose the ball*? *Can they keep control*?*”* (Timothy). Participants indicated this attribute may potentially discriminate players as the “*ability to receive a ball under pressure is important as this will highlight any deficiencies in their technique*” (Tyler) and is “*more important than technique in isolation*” (Campbell).

In relation to the identification of the important technical skills, participants indicated they assess this performance within a game or small-sided game environment, “*it is all related to the game*, *so the more you can create the game environment the better indication you will get*” (Tristan). Therefore, during the selection process recruiters use small-sided games to provide an indication of player’s technical skill ability, as “*less space to play*, *less time on the ball*. *So you are looking at how they react to those pressure situations”* (Carl). This is further explained by Tristan who stated, “*seeing them in 4 versus 4 to 9 versus 9 is enough to realise if their technique components are on the right pathway of development or nowhere near and it will take far too long for them to get to the right result*”.

Participants rated decision-making skills as most important tactical attribute with other tactical attributes such as game sense and awareness, anticipation, and general game understanding rated as moderately important. Decision-making was described as “*the ability of players to consistently make the correct decision after perceiving all the external factors and is fundamental to the game*” (Colin) and “*in a game situation are the players picking the right choices*?*”* (Timothy). Decision-making ability provides an indication of “*smart players who see things that not all players can see*” (Campbell) and can have a “*direct impact on quality of skill execution*” (Tristan). Game sense and awareness was described as “*the ability of the player to know what is going on around them*, *meaning they are constantly thinking about what will happen next rather than what is happening now”* (Carl) and this ability *“allows them to effectively execute core game skills”* (Tyson). This attribute was closely linked with the general game understanding attribute, with players expressing this attribute “*tend to display better decision-making*, *position and execution of actions”* (Chris). Furthermore, participants indicated anticipation was desired in players as *“it helps with the decision-making process and provides players within more time to execute in game actions”* (Campbell), and *“the ability to read the play and pre-empt opponents is a sign of an intelligent player”* (Thomas).

Tactical ability of players is assessed by, “*continuously putting players in game related situations*. *It might be 3 versus 2*, *4 versus 2*, *or 5 versus 3*” (Tyson). These situations provide players with the opportunity to “*look up*, *identify options and accurately play the best option*” (Tristan), with participants assessing whether “*they lose the ball or did they give it to the opposition*?*”* (Timothy). Further, tactical ability when the player is not in possession of the ball is also assessed, “*Where do they go*? *Some player’s go in the right places when their team has the ball they support in the right place*” (Thomas), with this providing an indication of decision-making ability as “*space gives you time to make a decision*” (Timothy). From a talent identification point of view, Travis indicated that you have to *“look at the intention*, *not necessarily the outcome*, *at this stage (of development)*. *So we base our selection on three things*: *perception*, *decision*, *and execution*”.

Participants also highlighted several psychological attributes important for skilled youth performance. Coachability and a positive attitude were ranked most important by the participants. Participants indicated players must demonstrate “*willingness to develop*” (Corey) as this potentially indicates they have “*a growth mindset and are willing to take new information and apply it*” (Colin). Participants specified that coachability is an important factor as if a player is not coachable, “*it limits their potential to improve*” (Tyler) and “*not worth spending the time and effort*” (Tyson). The identification of coachability in players involves the assessment of players actions outside of the competitive game environment, such as training or trial camps and is demonstrated in players who “*listen and whether or not they are prepared to ask questions in relation to what they don’t understand or don’t know*” (Chris).

Participants also indicated it is important for players to have a positive attitude, which may “*assist in their learning and development”* (Tyler). Furthermore, Thomas stated “*if you’re going to be a professional soccer player*, *you have got to have the right attitude*”. This right attitude was described as “*players demonstrating resilience*, *the ability to overcome adversities and not wanting to give up*” (Colin). Participants assessed positive attitude by “*watching them play and train*” (Thomas) and is demonstrated in the way they “*react after a mistake or setback in the game*” (Tait) because from the participant’s point of view “*it doesn’t matter how many mistakes you make*, *as long as the player is prepared to step up and go for the next action”* (Travis). Further, love of the game, confidence, competitive and personality/character were deemed moderately important for performance.

Physiological (e.g., strength, speed) and anthropometric (e.g., stature) attributes were excluded following the first round questionnaire. Participants indicated physiological attributes such as strength and speed are “*beneficial*, *but not essential for performance*” (Clive) and justified their as they “*can be developed later following growth spurts*” (Tyler). Further, “*every player develops differently and physical attributes are not something we are going to look for first and foremost*” (Terry), but rather identify players who are “*technically sound*” (Timothy). A potential reason for this emphasis on technical ability is the belief that “*It’s much harder to teach an eighteen or nineteen year old the technique and it just seems at that younger age*, *eleven through to fourteen*, *it is a real golden opportunity for their technique to start to improve*” (Connor). Interestingly, defensive ability was also deleted following the first round. The participants indicated their role is to “*develop a pro-active possession based game*” (Tyler) and whilst defending is an attribute of the game “*defending is an easier skill to coach than attacking options and can be developed with later coaching*” (Caden) and is therefore “*not a priority at this stage of development*” (Callum). Furthermore, some participants believed “*at this young age*, *most players lose the ball from mistakes rather than good defending from the opponent*” (Caden), therefore reinforcing the development of attacking based actions.

## Discussion

The current study was an innovative investigation describing the current talent identification practices of Under 13 representative team recruiters within Australia. The findings make a significant contribution to the current knowledge by providing initial evidence of the hierarchy of attributes Australian youth recruiters consider important when identifying and selecting Under 13 players for a representative team. The findings indicate technical (i.e., first touch, one-verse-one ability, and striking the ball), tactical (i.e., decision-making) and psychological skills (i.e., coachability and positive attitude) are perceived as most important within the talent identification process. In addition, recruiters also provided evidence of the attributes they do not consider important when identifying talented youth players for an entry level representative team, including physiological, anthropometric, and sociological qualities. Researchers have recommended coaches apply a holistic multidisciplinary approach to talent identification [3, 8], with the current findings potentially providing initial evidence to suggest that recruiters do consider numerous attributes when selecting and identifying youth players.

Recruiters perceived four technical skills (i.e., first touch, one-verse-one ability, striking the ball, and technique under pressure) as very important, with a players first touch the highest rated attribute. The justification for this high ranking was the belief that a player’s first touch is a foundation skill and the beginning of all other on-ball actions. Therefore, if a player has a limited or poor first touch, it can negatively impact the performance of all other on-ball technical skills, such as striking and running with the ball. Despite the importance placed on a player’s first touch by coaches there has been limited discussion within the literature regarding this technical attribute. Some researchers have provided evidence to suggest an isolated assessment of ball control, where a player has to keep the ball in the air without using their arms or hands, can differentiate skill levels [9, 14]. However, while this may provide an indication of the player’s ability to manipulate the ball with different parts of the body, a limitation of this assessment is the lack of ecological validity and limited relationship with a players first touch ability. The results from the current study suggest coaches and recruiters assess and identify players first touch ability via in-game or small-sided game performance. Therefore, to further improve current research knowledge, researchers need to consider how to develop in-game or small-sided game assessments of technical abilities to provide a more ecologically valid and practical assessment which may be used by coaches and recruiters.

The findings also suggest the perceived importance of other technical abilities such as striking the ball and one-versus-one interactions. Participants indicated that as soccer is a passing game players need proficient skills to be able to effectively and accurately distribute the ball and assist in attacking movements. While researchers have indicated isolated soccer-specific performance tests, including passing and shooting accuracy, whereby players were asked to hit a target from a designated distance, can differentiate skilled and less skilled technical performance [14, 22, 34], there has been limited exploration of methods to assess player’s one-versus-one ability. While previous investigations have assessed player’s technical ability to dribble the ball in isolated drills [14, 22, 27], these assessments lack ecological validity as players are required to dribble the ball around a set sequence of cones with no interaction with another player. Consequently, these isolated performance tests may not be beneficial for coaches or recruiters, with this supported by the participants in the current indicating they do not use isolated technical skill tests/assessments. Rather they indicated assessment of technical skills (i.e., striking the ball; one-versus-one ability) should be conducted within simulated game contexts, where manipulation of constraints creates an environment to assess specific technical skills. Therefore, the challenge for researchers is to create more ecologically valid assessments of technical abilities which can be implemented by coaches and recruiters.

The use of simulated games in the talent identification process also enables the examination of a player’s ability to perform technical skills in pressured situations. Participants suggested player’s technical ability must be robust enough to enable consistent performance during pressured situations. A further benefit of simulated games for assessment is the ability to identify decision-making skills of the players, as simulated games promote decision-making skills, which may be lacking within isolated soccer-specific performance assessments [35–36]. In relation to tactical skills, youth coaches perceived decision-making ability as very important for performance and game awareness, anticipation and general game understanding as moderately important. These results underline the importance placed on tactical skills at a youth level, as it can have an impact on skill execution. Therefore, youth recruiters believe that even at an Under 13 level, players need to be strong decision-makers to be identified as talented representative players. The findings from the current study corroborate with previous research which indicates decision-making performance can differentiate skilled and less skilled youth soccer players [10–12].

To assess tactical ability researchers have generally conducted video-based laboratory assessments with players making decisions relative to what on-ball action should be performed next (i.e., pass, dribble shoot) [10–12]. The results of the current investigation however suggest coaches and recruiters associate decision-making skills with both on and off ball actions (i.e., retain or lose possession; find space), and assess them within large and small-sided game situations. While this approach has been identified as an appropriate assessment method within the talent identification process [8], there is still limited understanding of how decision-making is assessed practically in-game and what coaches look for when judging decision-making performance. Therefore, the challenge for researchers is to further investigate the specific aspects of decision-making coaches and recruiters consider important to potentially develop objective assessments of youth player decision-making abilities within game environments.

The results also suggest coaches perceive some psychological attributes important when recruiting talented youth players, specifically coachability and a positive attitude. As indicated in the results, participants believe a willingness to learn new skills and a positive attitude to development is important in youth players. With respect to coachability, the descriptions provided by the participants in the current study align with the current literature definitions associated with coachability, including a player’s willingness to listen to the coach and a desire to learn new skills [37–38]. Players who were identified as coachable and have a positive attitude are more likely to have a growth mindset [39]. According to Dweck [39] players with a growth mindset have a commitment to learning, believe they can improve their ability with training and effort (it’s not innate), view mistakes as learning opportunities, are open to feedback and embrace challenges. Players who are deemed uncoachable or don’t have the right attitude may negatively impact team dynamics and performance [37, 40]. While coachability and positive attitude were rated highly by the coaches, there is still an underrepresentation of other psychological qualities such as motivation, resilience, commitment, and confidence which researchers have found to be predictors of soccer career success [5, 23–24]. Therefore, future investigations may consider the need to provide further education of these psychological qualities, or methods coaches and recruiters could use to assess them within potential youth players.

While the current study reported the attributes perceived as important for talented youth soccer players, the findings also highlight attributes recruiters perceive less important when selecting or identifying youth players. The extant literature recognises many physiological attributes which discriminate performance, with skilled youth players found to be faster, stronger, and more agile compared to less skilled players [1, 9–10]. Furthermore, Vaeyens and colleagues [1] found running speed to be the most important characteristic for Under 13 soccer players. The results of the current study however, suggest that while coaches believe speed and strength are beneficial, they do not perceive them essential when recruiting talented Under 13 players, but rather emphasis at this age group should be on technical ability. The recruiters justified their opinion by stating they believe speed and strength can be developed later in the player’s development, after their growth spurt, however it is much harder to teach technical skills to older players. This finding may be a reflection of the Football Federation Australia’s National Curriculum and their efforts to disseminate the current evidence regarding potential relative age effects. Furthermore, this supports previous findings within Australian youth soccer whereby there is evidence to suggest national youth team talent selectors are aware of relative age and maturity effects and do not base selection on age or physical maturity [41].

Interestingly, coaches also deleted defensive ability following the first round questionnaire. The justification for this was the participants believed this was also an attribute that can be developed later in a player’s development, and currently offensive player mistakes at this age group limit the need for exceptional defensive skills. Furthermore, during the interviews and the questionnaire process participants did not provide an indication of the potential sociological influences on player identification, such as parental support, education or cultural background. Researchers have indicated sociological factors are a potential predictor of talent in soccer and should be considered during the talent identification process [5, 8, 25]. Therefore, researchers should consider how sociological factors can be used in an applied setting by coaches and recruiters within the talent identification process.

While this is one of the first studies to understand what attributes youth coaches and recruiters perceive as important when identifying skilled youth performance at the entry level of representative soccer in Australia (i.e., Under 13 years), the findings should be considered with respect to several limitations. The findings provide the opinions, perspectives and ratings of key youth soccer performance attributes of an elite and experienced group of Australian soccer coaches and technical directors. While the participants were responsible for the selection of Australian regional representative team’s competing at the Under 13 national soccer championships, which is the highest level of competition for Under 13 players in Australia, their opinions and ratings may be biased by the Australian context. As a result, it may be possible that similar studies conducted in other countries may support or challenge the current reported findings. Therefore, whilst not an aim of this paper, future talent identification studies may consider cross-cultural or country analysis when attempting to identify key performance attributes. Also, the current study was limited by only reporting the attributes stated as being used by the experts, however it is possible that, in actuality, these attributes might not be used for talent identification. It is possible that some processes and attributes used to identify talent may not be available for conscious reflection and reporting (i.e., responses may be automatic or subconscious) and thus the rating of attributes (i.e., order of importance) may reflect attributes the participants think they should be using rather than attributes they actually use to make a decision. Therefore, while the current findings provide the initial step in identifying attributes used for talent identification, further research is needed to verify whether the reported attributes are used in the talent identification process and how. By gaining a better understanding of this process it may be possible to create more objective measures of performance within the talent identification processes.

## Conclusion

In soccer, talent selection and identification is important for the development of future elite level performers, with coaches continually seeking the attributes they believe predispose individuals for a successful soccer career [14, 27]. Researchers suggest coaches should apply a holistic multidisciplinary approach to talent identification [3, 8], and the current results may indicate that Australian youth coaches and recruiters consider a multidisciplinary approach to talent identification and selection. The findings provide initial evidence of the attributes Australian coaches consider important (i.e., technical, tactical and psychological) and also less important (i.e., physiological, anthropometrical, sociological) when identifying and selecting players for an Under 13 representative team. While the results demonstrate a more holistic approach by Australian coaches and recruiters in the selection of youth players, to improve our knowledge of the recruitment process researchers need to consider talent identification from a holistic rather than the current isolated approach. This may then provide more understanding of how coaches and recruiters identify talent, or use certain game situations to make subjective judgements on players, in an attempt to create more objective instruments or testing procedures which may clarify this process for all key stakeholders.

## Supporting information

S1 File Questions for the interviews. (DOCX)

S1 Dataset Minimum data set. (XLSX)
