# Supplementary material for: Talent identification and recruitment in youth soccer: Recruiter’s perceptions of the key attributes for player recruitment
Source: PLoS One. 2017 Apr 18;12(4):e0175716. doi: 10.1371/journal.pone.0175716 (PMC5395184; doi:10.1371/journal.pone.0175716)
Supplement: S1 File — (DOCX) [file pone.0175716.s001.docx]

**Key Attributes for Elite Youth Soccer Players.**

**Semi-Structured Interview Questions**

1. When you are identifying talent at an under 13 level what is it you are looking for?
   1. physiological, anthropometric, technical, tactical, psychological, decision-making
   2. Why do you rate this attribute?
   3. How do you identify it in a player?
   4. Why is this attribute important for players of this age?
   5. Can coaches develop this attribute if a player is selected without this attribute?
2. Do you believe there are some attributes that are more important to players of this age than others?
3. How do you decide between a talented or less talented player at this age group?
4. For you, what are the initial signs of a player at this level who may make it as a player?
5. What are the challenges to players of this age making it as a professional/elite/senior player?
6. Can you outline the selection process you undertake when selecting players of this age?
   1. Trials open or invited?
   2. Competitive games? Training activities or games?
   3. Do you incorporate any testing strategies?
   4. Is there a selection criteria
   5. Timeframe of the process
